# Towards a cancer mission in Horizon Europe: recommendations

**DOI:** 10.1002/1878-0261.12763

**Published:** 2020-08-04

**Authors:** Anton Berns, Ulrik Ringborg, Julio E. Celis, Manuel Heitor, Neil K. Aaronson, Nancy Abou‐Zeid, Hans‐Olov Adami, Kathi Apostolidis, Michael Baumann, Alberto Bardelli, René Bernards, Yvonne Brandberg, Carlos Caldas, Fabien Calvo, Caroline Dive, Angelika Eggert, Alexander Eggermont, Carolina Espina, Frederik Falkenburg, Jérôme Foucaud, Douglas Hanahan, Ulrike Helbig, Bengt Jönsson, Mette Kalager, Sakari Karjalainen, Miklós Kásler, Pamela Kearns, Klas Kärre, Denis Lacombe, Francesco de Lorenzo, Françoise Meunier, Gerd Nettekoven, Simon Oberst, Péter Nagy, Thierry Philip, Richard Price, Joachim Schüz, Eric Solary, Peter Strang, Josep Tabernero, Emile Voest

**Affiliations:** ^1^ The Netherlands Cancer Institute Amsterdam the Netherlands; ^2^ European Academy of Cancer Sciences Stockholm Sweden; ^3^ Cancer Center Karolinska Karolinska University Hospital Stockholm Sweden; ^4^ Danish Cancer Society Research Centre Copenhagen Denmark; ^5^ Ministry for Science, Technology and Higher Education Lisbon Portugal; ^6^ Fondation ARC pour la recherché sur le cancer Villejuif France; ^7^ Karolinska Institutet Stockholm Sweden; ^8^ European Cancer Patient Coalition Brussels Belgium; ^9^ German Cancer Research Center (DKFZ) Heidelberg Germany; ^10^ The European Association for Cancer Research Nottingham UK; ^11^ Department of Oncology University of Torino Candiolo (TO) Italy; ^12^ Cancer Research UK Cambridge Centre Cambridge UK; ^13^ Gustave Roussy Cancer Campus Grand Paris Villejuif France; ^14^ Cancer Research UK Manchester Institute The University of Manchester Manchester UK; ^15^ Charite‐Universitatsmedizin Berlin Germany; ^16^ Princess Máxima Center for Pediatric Oncology Utrecht the Netherlands; ^17^ International Agency for Research on Cancer (IARC/WHO) Lyon France; ^18^ Cancer Prevention Europe Lyon France; ^19^ Dutch Cancer Society Amsterdam the Netherlands; ^20^ French National Cancer Institute (INCa) Boulogne Billancourt France; ^21^ Swiss Institute for Experimental Cancer Research (ISREC) Federal Institute of Technology in Lausanne (EPFL) Lausanne Switzerland; ^22^ Swiss Cancer Center Leman (SCCL) Lausanne Switzerland; ^23^ German Cancer Aid Bonn Germany; ^24^ Stockholm School of Economics Stockholm Sweden; ^25^ Institute of Health and Society University of Oslo Oslo Norway; ^26^ Association of European Cancer Leagues (ECL) Brussels Belgium; ^27^ Ministry of Human Resources Budapest Hungary; ^28^ SIOPE Head Office Brussels Belgium; ^29^ NIHR Birmingham Biomedical Research Centre University of Birmingham Birmingham UK; ^30^ The Swedish Cancer Society Stockholm Sweden; ^31^ EORTC Headquarters Brussels Belgium; ^32^ Federation of European Academies of Medicine Brussels Belgium; ^33^ Organisation of European Cancer Institutes (OECI) Brussels Belgium; ^34^ National Institute of Oncology Budapest Hungary; ^35^ Institut Curie Paris France; ^36^ The European Cancer Organisation (ECCO) Brussels Belgium; ^37^ Vall d’Hebron University Hospital and Institute of Oncology (VHIO) Barcelona Spain; ^38^ Cancer Core Europe Amsterdam the Netherlands

**Keywords:** cancer mission, cancer research/care/prevention continuum, comprehensive cancer centres, European healthcare systems, patient empowerment, science policy

## Abstract

A comprehensive translational cancer research approach focused on personalized and precision medicine, and covering the entire cancer research–care–prevention continuum has the potential to achieve in 2030 a 10‐year cancer‐specific survival for 75% of patients diagnosed in European Union (EU) member states with a well‐developed healthcare system. Concerted actions across this continuum that spans from basic and preclinical research through clinical and prevention research to outcomes research, along with the establishment of interconnected high‐quality infrastructures for translational research, clinical and prevention trials and outcomes research, will ensure that science‐driven and social innovations benefit patients and individuals at risk across the EU. European infrastructures involving comprehensive cancer centres (CCCs) and CCC‐like entities will provide researchers with access to the required critical mass of patients, biological materials and technological resources and can bridge research with healthcare systems. Here, we prioritize research areas to ensure a balanced research portfolio and provide recommendations for achieving key targets. Meeting these targets will require harmonization of EU and national priorities and policies, improved research coordination at the national, regional and EU level and increasingly efficient and flexible funding mechanisms. Long‐term support by the EU and commitment of Member States to specialized schemes are also needed for the establishment and sustainability of trans‐border infrastructures and networks. In addition to effectively engaging policymakers, all relevant stakeholders within the entire continuum should consensually inform policy through evidence‐based advice.

## Graphical abstract

A comprehensive cancer approach covering the entire research–care–prevention continuum can achieve a 10‐year cancer‐specific survival for 75% of patients diagnosed in EU member states with well‐developed health care by 2030. To ensure access to a critical mass of patient biological and technological resources, infrastructures for translational research, clinical and prevention trials, and outcomes research are needed. Here, we provide recommendations for achieving key targets and prioritize research areas.

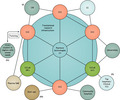

**Abbreviations**
AI: Artificial intelligence
CCC: Comprehensive Cancer Centre
CCCoE: Comprehensive Cancer Centre of Excellence
CEEAO: Central‐Eastern European Academy of Oncology
CPE: Cancer Prevention Europe
EACR: European Association for Cancer Research
EACS: European Academy of Cancer Sciences
EC: European Commission
ECPC: European Cancer Patient Coalition
EMBO: European Molecular Biology Organization
EORTC: Organisation for the Research and Treatment of Cancer
ESMO: European Society for Medical Oncology
ESSO: European Society of Surgical Oncology
ESTRO: European Society Radiotherapy and Oncology
EU: European Union
OECI: Organization of European Cancer Institutes
SIOPE: European Society of Paediatric Oncology

## 1 Introduction

Recently, the European Academy of Cancer Sciences (EACS) and several European organizations and cancer centres joined forces to define common goals for the implementation of a mission‐oriented approach to cancer in Horizon Europe, initially proposed by Celis and Pavalski in 2017 [1, 2, 3]. The aim is ‘to have an impact on society at large by uniting countries to substantially reduce the enormous cancer burden in the European Union (EU) and improve the health‐related quality of life of patients by promoting cost‐effective, evidence‐based best practices in cancer prevention, treatment, and care’. As highlighted previously, the main goal is to ‘achieve a 10‐year cancer‐specific survival for ¾ of the adult patients diagnosed in year 2030 in Member States with a well‐developed healthcare system. Because cancer mortality provides a timelier assessment of progress also capturing advances in both therapeutics and prevention, it will be important to document the expected declining trends of age‐standardized mortality in each EU country’ [1, 2]. The objectives of the mission must be mindful of the needs of the European patients and citizens at large, by bringing maximum value for public investment, and to ensure that health technologies developed by funding through the mission are available to those who need them for a fair and affordable price.

This goal can only be achieved by integrating and bridging the entire continuum of cancer research, prevention and care, which spans from basic, epidemiological and preclinical research to clinical, prevention, implementation and survivorship research. Particular attention should be paid to the gap between research and cancer care, and research and prevention. Different disciplines are involved in this endeavour each with their own specific emphasis. These include the following: (a) cancer biology (basic and preclinical research); (b) identification of healthy individuals at risk of developing cancer (primary prevention); (c) early cancer detection (secondary prevention); (d) cancer patient treatment and research (clinical); and (e) support for cancer survivors (tertiary prevention). Assessing progress in these areas requires different methodological approaches [4, 5, 6]. Outcomes research for both therapeutic interventions and the effectiveness of public health interventions and health services will be critical for progress assessment. This will require adequate resources, multidisciplinary expertise, access to large, high‐quality data sets including patient records, suitable analysis tools and coordinated collaborative projects. Taken together, all the above elements are essential for achieving science‐driven medical and social innovations and their resulting intervention trajectories, all tailored to the individual needs of patients [2].

The latter goal emphasizes the need to create integrated, networked and geographically distributed infrastructures that can entail Comprehensive Cancer Centres of Excellence (CCCoEs) meeting the Excellence standards of the EACS [7], Comprehensive Cancer Centres accredited by the Organisation of European Cancer Institutes (OECI), cancer research and clinical centres and technological platforms. CCCs are crucial to establish closer links between research and healthcare systems [8, 9]. By integrating cancer care and prevention with research and education, CCCoEs and CCCs are well‐positioned to boost innovation and deliver state‐of‐the‐art comprehensive multidisciplinary cancer care. Only a few designated CCCs do incorporate paediatric care as paediatric cancer patients often also require specific expertise only available in children hospitals. Nevertheless, further concentrating paediatric oncology in centres with the necessary critical mass can boost innovation and effectiveness of the treatment of children with cancer. Across Europe, the integration of cancer research and clinical care for children and adolescents needs to address the exquisite circumstances of this patient population, as has been demonstrated in the successful launch of the European Commission (EC) supported Paediatric Cancer Expert Reference Network (https://paedcan.ern‐net.eu/). Geriatric patients, which constitute a much larger group, are best served by CCCs that have specific programmes focussed on the specific needs of elderly patients.

In this update, which accommodates the input of many European cancer organizations, we provide a more detailed view of the infrastructural requirements to promote excellence in cancer research. We also emphasize consensus priority areas to realize the cancer mission objectives and outline recommendations for engaging professionals and institutions throughout Europe.

## 2 Infrastructures to support cancer research of excellence

We want to emphasize that creativity, originality, curiosity and a visionary foresight among individual scholars or teams of investigators remain the engine for innovation and discovery. However, these investigators need to be embedded in infrastructures of sufficient critical mass. This is essential for effectively linking basic, translational, clinical and prevention cancer research with care, as well as for driving innovation across the whole cancer research/care/prevention continuum. Such infrastructures would provide researchers access to essential technology platforms, resources and patients.

**Fig. 1:**
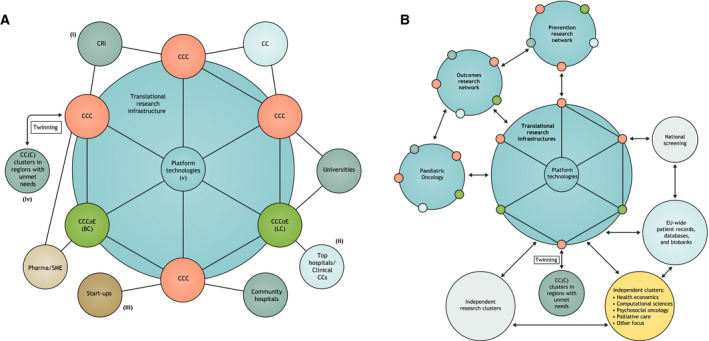
Research networks provide cancer researchers with sufficient critical mass of research infrastructures, patients, samples, technology and expertise. (A) Paradigm of a translational research network. Multidisciplinary, patient‐centred institutions, such as CCCs and CCCoEs, each having a broad research scope, interact closely. For example, they collaborate on specific research items (indicatively, on breast cancer (BC), or lung cancer (LC)) and share platform technologies, thereby forming the core components of a translational research infrastructure. CCCs and CCCoEs are best positioned to: (i) support basic and translational research through crosstalk with cancer centres (CCs) and cancer research institutes (CRIs), as well as linking to academic research at universities, for example, research on (bio)chemistry, engineering, genetics, molecular and cell biology, tumour biology, immunology; (ii) exchange data to improve care for patients both at top clinical hospitals and community hospitals; (iii) work closely with start‐ups and offer pharmaceutical and biotechnology industries strategic partnerships; (iv) provide training, capacity building and mobility of researchers and clinicians across Europe through twinning programmes; (v) generate intellectual property and engage in profitable technology transfer, facilitating the communication and dissemination of information. (B) Infrastructures involve interacting networks. These networks too are based on the close collaboration among researchers in CCCs, CCCoEs, clinical CCs, universities and other research organizations (see also panel A). The three suggested types of infrastructures (translational research, clinical and prevention trials, and outcomes research) may in addition include structures addressing specific research requirements. An already‐established paediatric oncology network exemplifies how innovative research and clinical strategies can be delivered, based on strong collaboration across European centres. In the context of a cancer mission, all networks would establish cross‐border relationships with each other, and also with existing independent research clusters and professional clusters focusing, for example, on health economics, computational sciences, psychosocial oncology or palliative care. In addition, strong links to national screening facilities, and EU‐wide patient records, databases and biobanks can be established and maintained.

Multidisciplinary/professional patient‐centred institutions are best positioned to (a) support basic and translational research, (b) link research with the healthcare systems including prevention organizations, (c) offer pharmaceutical and biotechnology industries strategic partnerships, (d) generate intellectual property and engage in profitable technology transfer, (e) provide training, capacity building and mobility of researchers and clinicians across Europe, (f) facilitate the communication and dissemination of information and finally (g) provide the best care for patients (Fig. 1A). Networks of such institutions, accessible to research teams across Europe, will be essential to achieve the goals. Specialized academic medical/cancer research centres are critical especially for primary prevention research and intervention research [10]; their particular target population of healthy individuals and their research often based on observational rather than intervention studies, with links to basic research, epidemiology, public health and social and human sciences.

We propose three networked research infrastructures accessible to research teams from across Europe that will be essential to achieve the goals. The three infrastructures should focus on translational research, clinical and prevention trials, and outcomes research (Fig. 1B).

### 2.1 Infrastructure for translational research

Translational research bridges basic/preclinical research with clinical and prevention research, builds on inventions and innovation from basic/preclinical research, and has a direct impact on therapeutic and prevention research [1, 3]. This should result in proof of principle clinical/prevention trials that, if successful, subsequently require research for effective implementation in the healthcare system.

A comprehensive infrastructure for translational research linked to clinical research will require:

- A robust basic cancer research programme.
- Close interactions between innovative basic/preclinical research, molecular and digital pathology, a variety of omics technologies and immunotyping facilities for patient stratification.
- A bidirectional translational research structure.
- Data acquisition tools and structured databases with possibilities for computational analyses relevant for both therapeutics and prevention studies.
- Reduced fragmentation of oncology data sources through a well‐functioning European Health Data Space with focus on the integration of real‐world data sources and harnessed quality‐of‐life data; data safety, open science and FAIR principles (findable, accessible, interoperable and reusable). Harmonized interoperability standards, data sharing.
- Innovative imaging technologies, with a focus on novel molecular and functional imaging.
- Facilities and expertise to develop and implement cell‐based and other biological therapies.
- High‐quality pharmacology.
- Biobanks with associated patient records.
- Capacity for ‘proof‐of‐concept’ clinical/prevention trials.
- Longitudinal sampling routines (tumour biopsies/consecutive biopsies and liquid biopsies).
- Interaction with clinical‐trials consortia or networks to develop practice‐changing clinical trials.

### 2.2 Infrastructure for clinical and prevention trials

‘Proof‐of‐principle’ studies may serve as a starting point for further clinical and prevention research, including the assessment of its utility in health care or prevention, and patient‐reported outcomes. Well‐developed clinical trial structures, as well as advanced diagnostic methods, such as state‐of‐the‐art molecular pathology, omics technologies and pharmacology to stratify patients, are crucial.

Due to a large number of tumour subgroups, the traditional clinical trials methodology built on the phase I–IV trial concept are gradually being superseded by new more sophisticated stratification methods [11, 12, 13, 14]. Moreover, there are increasing possibilities to follow therapy response using innovative imaging technologies, consecutive tumour biopsies and/or liquid biopsies [15]. Such biopsies permit treatment adjustment to the changing biology of the tumour. However, it will be essential to monitor closely whether these more advanced and potentially costly interventions improve patient outcome; implementation research can determine this.

Implementation research needs to include health economics of therapeutic interventions and prevention programmes for early detection on large patient populations, to inform on their clinical utility, benefits and harms to patients and the healthcare system at large. In addition, patients' experiences during new treatment approaches have to be considered. The patient's gender and age are also parameters that need to be carefully weighed in clinical trial designs. Paediatric oncology is an obvious example, but this equally holds for elderly patients. Given the ageing population, age may be considered as an essential parameter in the implementation (adaptation of therapeutic strategies, importance of supportive care, presence of comorbidities and frailties) and evaluation (health‐related quality of life) of clinical and prevention trials.

By contrast, as primary prevention mostly addresses harmful exposures and behaviours, research is observational and often requires hundred thousands of individuals in multinational study series to draw firm conclusions. Implementation of protective measures and secondary prevention effectiveness and efficacy can be evaluated in field trials, with the individual or sometimes even communities, serving as observational units [5]. Tertiary prevention, although involving the cancer patient, usually follows the individual well beyond the time they are in contact with a cancer hospital.

A comprehensive infrastructure for clinical and prevention trials will require*:*

- Availability of sufficiently large numbers of diverse patient groups for clinical research to develop personalized/precision cancer medicine, in case of prevention trials access to large numbers of healthy subjects.
- Molecular pathology including multi‐omics technologies and immunotyping for stratification of patients and healthy subjects for distinct treatment arms.
- State‐of‐the‐art infrastructure for early clinical trials, next‐generation clinical trials, practice‐changing clinical trials and implementation research.
- Follow‐up monitoring/treatment adaptation by repeated biopsies and functional/molecular imaging technologies.

**Fig. 2:**
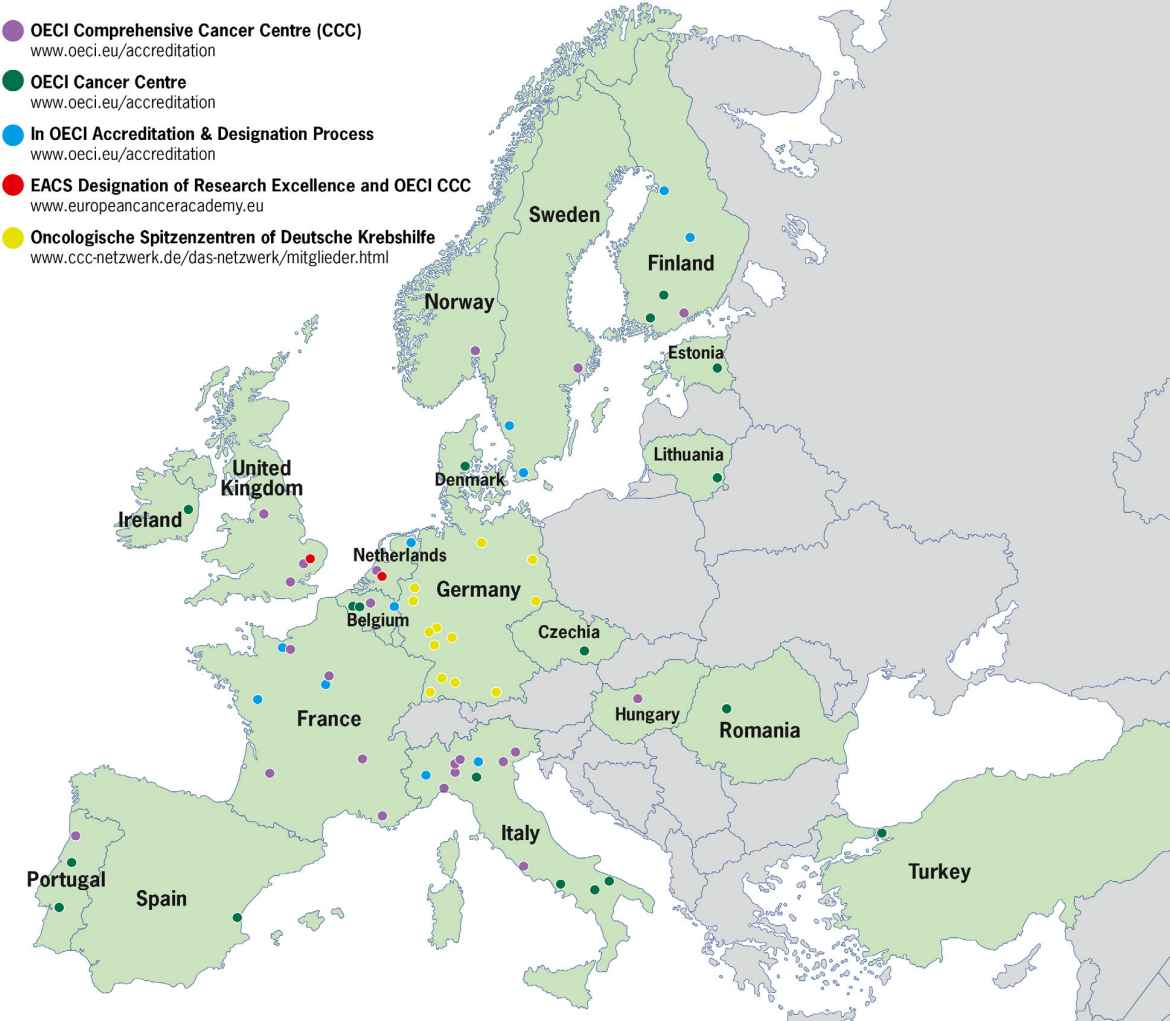
Overview of Accredited CCCs and Cancer Centres in Europe.

Comprehensive cancer centres and CCCoEs (Fig. 2) often fulfil many of these requirements as far as the clinical trial trajectory is concerned. They are further complemented with clinical research networks, many in collaboration with Organisation for the Research and Treatment of Cancer (EORTC), an organization that will play a significant role in this infrastructure. However, CCCs currently lag behind in implementation research, which we consider an essential aspect that needs to be addressed. Prevention research is not sufficiently covered in most CCCs and also requires distinct infrastructures which might vary depending on the nature of the trial. Clearly, it has to include strong epidemiology, biostatistics, data acquisition capacity and advanced computational capabilities. IARC fulfils a critical international role in this latter domain, and Cancer Prevention Europe (CPE) is expected to make critical EU‐focused contributions.

### 2.3 Infrastructure for outcomes research

Evidence of the effectiveness of therapeutics and prevention strategies is essential for the assessment of clinical utility, cost‐effectiveness and prioritization [16]. In addition to showing effectiveness in clinical and prevention trials, evidence of effectiveness in day‐to‐day clinical practice is required. For therapeutics, data from quality‐assured clinical registries are indispensable for evaluating effectiveness. Outcomes research in therapeutics addresses questions related to all aspects of the clinical pathway, including treatment optimization, side effects of treatments, long‐term follow‐up with assessment of health‐related quality of life, rehabilitation and survivorship, as well as attention to social aspects. This should preferably be a collaborative effort between clinicians, researchers and epidemiologists. For prevention, outcomes can be measured using data from population‐based registries for cancer incidence and mortality.

Areas of research that need special attention for patients living with cancer are rehabilitation, psycho‐oncology, sequelae prevention and supportive care for palliative oncology, as well as survivorship [17]. Since around half of all cancer patients in the EU will ultimately need palliative care (nearly all patients that die from cancer), this area requires specific attention. Outcomes research has to differentiate between: (a) supportive care when cure is no longer possible but life extension with a good health‐related quality of life still is a reasonable goal and (b) palliation at the end of life. Patients with rare cancers and in specific vulnerable age ranges, that is, children and the elderly will need more tailored regimes.

A network of CCCs with consistently structured clinical registries will be instrumental for collecting the necessary data and formulating research questions. By stimulating collaborations between CCCs, the critical mass will be in place for effective outcomes research. The EACS plans together with OECI to identify the criteria for designation of CCCoEs and CCCs, which will be instrumental for Outcomes Research.

A comprehensive infrastructure for outcomes research will require:

- Extensive collaboration among clinicians, epidemiologists and other researchers in CCCoEs, CCCs, clinical cancer centres, universities and other research organizations. Use of networks within networks, an infrastructural model designed by Cancer Core Europe, will be essential for high‐quality outcomes research.
- Competencies in epidemiologic theory, biostatistics, bioinformatics, artificial intelligence (AI) — including big data and machine learning —as well as communication technology, which should become an integrated part in many aspects of cancer research, treatment and prevention.
- Well‐structured databases with preclinical, clinical and socio‐economic data, and data from observational studies (patient registries/databases). These databases, preferentially deposited in EU‐controlled data centres, should allow linkage to randomized data platforms. Eligible patients can be invited to participate. State‐of‐the‐art computational tools need to be linked to the databases.
- Pan‐European databases on patients with rare cancers. Outcomes research on rare cancers is difficult to achieve in individual countries due to the limited number of cases. The European Reference Networks can play here an important role (https://ec.europa.eu/health/ern/networks_en ).
- Complete and updated national cancer registries. The NORDCAN database provides an example of easily accessible data on cancer incidence and death (https://www‐ep.iarc.fr/NORDCAN/English/frame.asp ).
- Comprehensive and updated national cause of death registries.
- Transparent data‐sharing policies. This is a critical prerequisite to perform effective outcomes research.

### 2.4 Infrastructure models

The models of the infrastructures suggested above can be based on the structures of some existing networks and some key recommendations listed below. CCCs such as those accredited by the OECI [9], the German Cancer Aid [18], or CCCoEs designated by the EACS with focus on translational research [7] will be key components of the three infrastructures. These centres are well‐positioned to form networks, both nationally and internationally, and some indeed have done so, both within and beyond national boundaries. Networks composed of CCCoEs, CCCs, cancer research institutes and clinical centres with well‐developed integrated basic, preclinical and clinical research, as well as relevant technical platforms, will be important elements of the infrastructures (Fig. 1B). Institutional collaborations will enable the recruitment of sufficiently large patient cohorts, access to biological materials and technological resources, as well as the establishment of sustainable large‐scale research programmes. Cancer Core Europe is an example of a translational cancer research consortium for therapeutics [19, 20, 21], and CPE an example of a network for prevention research [5, 22]. These consortia share common interests, and their close interaction will be crucial to explore the biology underlying known and new causes of cancer. Such interactions can result in new prevention programmes and diagnostic technologies to detect malignant disease at an early stage, thereby permitting treatment that is more effective.

The network model of infrastructures adopted by Cancer Core Europe is based on institutional collaborations (legal entity) among seven large cancer centres across Europe, most of which are CCCs [19, 20, 21]. The German Cancer Research Consortium (DKTK) is a national entity linking eight CCCs; moreover, within the frame of the German National Decade against Cancer, a German consortium of six National Centres for Tumour diseases is under development to structure the clinical part of the research continuum as well as a National Cancer Prevention‐Development Strategy. Another prime example of a national network is the Cancer Research UK network of 15 translational research centres, which are funded to the tune of €230 million a year (https://www.cancerresearchuk.org/funding‐for‐researchers/our‐research‐infrastructure/our‐centres) on top of competitive grant funding. A further example of a national legal entity linking 20 Cancer Centres in France is Unicancer (http://www.unicancer.fr/en/patients/unicancer‐charter ) furthering translational and clinical research, and clinical improvements. An additional example is the collaborative initiative taken by paediatric oncologists through the European Society of Paediatric Oncology (SIOPE; https://siope.eu/encca/).

### 2.5 Recommendations for creating the three types of infrastructures for innovative cancer research

#### 2.5.1 Create networks of CCCs and CCCoEs

Today around 35 CCCs are accredited in Europe, 22 by the OECI and 13 by the German Cancer Aid; two CCCoEs are already certified by the EACS (Fig. 2). It is essential to have at least one CCC in each country acting as a nucleus from which expertise and best practices are disseminated within the country, and some larger Member States might need 10 or more CCCs. Newly accredited CCCs, generated through supportive partnership arrangements, should result in networks of CCCs/CCCoEs and other centres (both within Member States and across borders) to innovate and perform high‐quality multidisciplinary cancer research and provide high‐quality cancer care, including health‐related quality of life and survivorship research. They may also conduct prevention research and offer prevention services depending on how health care is organized in each country. CCCoEs, on the other hand, should provide advanced infrastructural facilities. To increase the number of CCCs, institutions that have capabilities to become a CCC or an accredited clinical centre need to be incentivized by establishing funding opportunities to reach the standards required for formal accreditation by the OECI [23]. Countries that do not have CCCs are recommended to establish at least one CCC, through appropriate funding instruments (e.g. cohesion funds).

#### 2.5.2 Generate incentives for ‘twinning’ a CCC or clinical centre with an established CCCoE or equivalent high‐quality centre, to facilitate the training of specialists and researchers

The aim is to increase the knowledge and skills of cancer professionals and to promote research collaborations, thereby boosting healthcare innovation (Fig. 1). ‘Twinning’ could be initiated from clinical centres (or individuals working in these locations) that aspire to accreditation, or from established CCCs that want to reach out to raise the standards of centres elsewhere. The funding mechanism should be flexible and avoid unnecessary bureaucracy. Examples are already in place: The German Cancer Research Centre (DKFZ, Heidelberg) has twinned with the Athens CCC (http://www.accc.gr/), and the Swedish Karolinska Institute (KI, Stockholm) is in discussion about expanding an existing formalized collaboration with the National Institute of Oncology NIO, Budapest, into a twinning partnership (https://onkol.hu/kutato‐osztalyok/?lang=en and https://onkol.hu/department_of_selenoprotein_research/?lang=en ). Experiences acquired through these collaborations could help the development of new ‘twinnings’. Cancer Core Europe is supporting this development, and the engagement of the recently established Central‐Eastern European Academy of Oncology (CEEAO) could play a strategic role in this endeavour (https://hungarytoday.hu/kasler‐central‐eastern‐european‐academy‐of‐oncolog/). The OECI, the EACS and the European Association of Cancer Research (EACR) will be of critical importance in the areas of training and education.

To make an impact, however, the infrastructures need to be sustainable. Only then, a number of such collaborative entities can be created and the inclusion of institutions in all EU Member States secured. The ERA‐NET TRANSCAN (https://www.era‐learn.eu/network‐information/networks/transcan‐2), for example, offers a strategy to support international translational cancer research collaborations and will greatly benefit from the proposed infrastructures. Developing and expanding infrastructures will require open access to knowledge, transparent access rules to data, commitment from all Member States, alignment of European and national funding sources, as well as instalment of strong governance and management.

## 3 Research portfolio: areas of priority

The cancer mission aims to apply and expand present knowledge to reduce cancer incidence and mortality and to improve health‐related quality of life by promoting affordable, evidence‐based best practices in cancer prevention, treatment and care. Coordinated multidisciplinary research in the consensus areas highlighted below, supported by the networked infrastructures described above, will be necessary to achieve the mission goals.

### 3.1 Basic and preclinical research

Basic research is essential to enlighten our understanding of the molecular mechanism underlying cancer [24] and is the engine that fuels innovation in both prevention and therapeutics [1, 25]. Our recommendations (Box 1) may help maximize the potential of basic and preclinical research which provide the basis for speeding up the translation of discoveries into clinical and potentially preventive applications that impact patients' lives and benefit society at large.

Box 1 **Recommendations for basic and preclinical research.**

- Encourage multidisciplinary projects (cancer biology, chemistry, immunology, radiobiology, engineering, computational science, public health).
- Promote high risk–high return projects.
- Promote research in poor prognosis cancers.
- Engage researchers from all EU countries.
- Use ERC funding paradigms to select the most promising bottom‐up proposals.
- Facilitate participation of small and medium enterprises and industry.

A number of research areas are expected to have a bearing on the innovation of prevention and therapeutics research. Research towards identification of new causes of cancer through unravelling mechanisms of carcinogenicity, the biology underlying premalignant and malignant lesions, identification and validation of biomarkers for detecting premalignant disease, and elucidation of the role of ageing and comorbidities in the emergence and progression of malignant clones is expected to result in new preventions strategies. In addition, for development of therapeutics with a focus on medical oncology, the following are vital: prediction of antitumour effects and side effects of treatment; development of technologies to stratify patients for treatment; innovation of precision pharmacological monitoring; mechanisms underlying drug and immunotherapy resistance, and how to overcome them; as well as characterization and manipulation of the tumour microenvironment. Innovation in imaging and radiation therapy is dependent on basic/preclinical research [26]. Involvement of computational sciences will gain more in‐depth insight into cancer biology and clinical/prevention cancer research.

### 3.2 Primary prevention

**Fig. 3:**
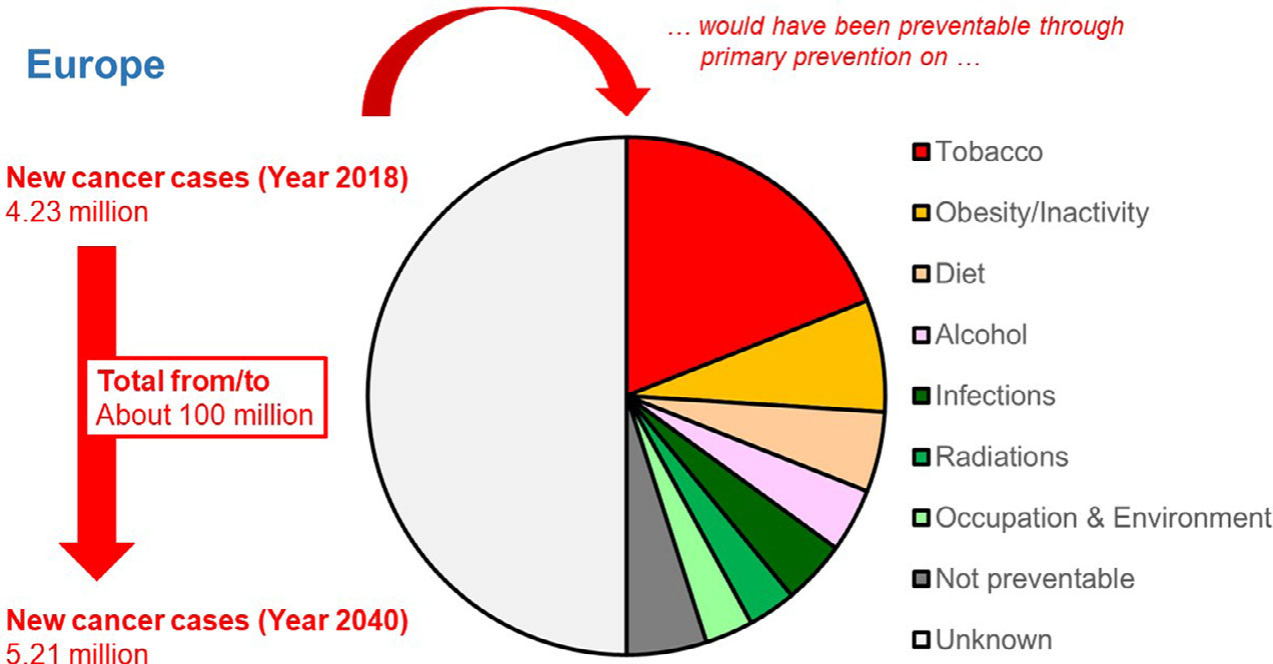
Newly diagnosed patients with cancer estimated for the year 2018 and projected for the year 2040 for Europe (UN definition), the predicted new cancer burden for the total period from 2018 to 2040, and the preventable cancer burden in 2018 had primary prevention against the listed established causes of cancer been rigorously implemented [22] (Source: J. Schüz—Modifiable risk factors and prevention: overview of current knowledge and main challenges; European Code against Cancer initiative. Health Working Group; Environment, Public Health and Food Safety (ENVI) Committee of the European Parliament, 18/2/2020: https://www.europarl.europa.eu/cmsdata/196417/Schuz_modifiable%20risk%20factors.pdf).

Primary prevention research has provided recommendations to decrease, for example, tobacco smoking, alcohol consumption and exposure to UV radiation by the sun or UV devices and to maintain a normal body weight [27]. Figure 3 puts the preventable fraction of cancers through primary prevention in the context of the increasing European cancer burden. However, implementation is often inadequate [5]. For some preventive measures known to be successful, there are political and societal barriers delaying or even hampering implementation; notably, cigarette smoking remains responsible for almost half of all preventable cancer cases in Europe [22]. For many other known harmful exposures or unhealthy behaviours, the most effective and efficient preventive strategies are not yet identified. Consequently, implementation research is essential to augment the effectiveness of such programmes. Such research should address awareness in society, particularly concerning attitudes and lifestyles, as well as the role of authorities in regulating the consumption of harmful substances and exposure to environmental carcinogens. Additional research areas, such as public health, sociology, and behavioural science, have to be integrated into this research. If behavioural change is the goal of the preventive measures, it is essential to include expertise in these areas (Box 2).

Box 2 **Recommendations for primary prevention.**

- Support implementation research to enhance the effectiveness and efficacy of prevention programmes that address well‐known risk factors (tobacco, UV exposure, alcohol consumption, overweight) and if effective would substantially reduce cancer incidence throughout the EU.
- Support continued aetiological research to uncover new causes of cancer, genetic predisposition and the influence of behavioural and environmental factors.
- Support population health intervention research to develop operational strategies and policies in cancer prevention, for example new primary prevention strategies (vaccination, medical) that are less expensive and easy to implement, independent from the expenditure on health
- Promote research to elucidate the individual and societal cognitive processes behind successful behavioural preventative interventions and to address the socio‐economic and commercial determinants of health.care in a particular country.
- Promote behavioural/nudging, area‐based/territory‐based/community‐based intervention research linked to prevention, by engaging scientists from disciplines less represented in cancer research today, such as behavioural, communication and social sciences.
- Overall funding for prevention research must increase substantially, and new areas of research must be included.

Prevention research should involve identification of causes of cancer and individuals at high risk (exposure and genetic predisposition) using epidemiological research coupled with mechanistic studies, including interactions of risk factors. Research is needed to reduce carcinogenic exposures (environment, workplace) and addiction to carcinogenic substances as well as to uncover underlying biological and social mechanisms. Behavioural research linked to changing lifestyle patterns that increase cancer risk and long‐term side effects of treatment and offering active primary prevention (e.g. vaccination, novel targets for medical prevention) is other relevant research areas. Implementation research should be structured and optimized (Box 2).

### 3.3 Early detection for prevention and treatment

The distinction between early benign disease and premalignant disease likely progressing to invasive/metastatic disease is still difficult [28]. Identification of early markers, obtained from early lesions or liquid biopsies, that predict progression to malignant disease, will be extremely valuable for effectively eliminating malignant disease early. In addition, combining early detection with the identification of individuals at high risk, based on lifestyle and/or genetic predisposition, will enhance the innovation and effectiveness of screening and early detection programmes [29]. Programmes of early detection will be critical particularly within primary‐ and community care, linked to the expertise and data in specialist centres within networks. The impact of the COVID‐19 crisis has already shown large falls in symptomatic presentations to primary care, and screening [30]; (https://www.bbc.com/news/health‐52985446 ). This fragility points to the need for targeted presymptomatic interventions offered to individuals based on risk profile.

Significant research initiatives are already devoted to identify the specific characteristics of early lesions and to develop new diagnostic methods [31]. However, much remains to be learned, and it will require substantial efforts to develop valid predictive diagnostic assays for early detection of malignant disease. Once promising methods are available, well‐structured implementation research will be needed to evaluate their effectiveness in screening programmes. Assessment of clinical effectiveness combined with health economics is critical. The outcome of early detection and treatment has to be compared to the outcome of treatment following manifestation of clinical symptoms. This type of information will be necessary to prioritize early detection programmes within the EU and to assure that the most effective screening programmes are rolled out first. In addition, swift access to medical care is essential for individuals experiencing symptoms that warrant further examination. More research on the impact of healthcare systems on early detection is needed. We also need effective approaches to make the population more aware of early signs of disease. Our recommendations for early detection are summarized in Box 3.

Box 3 **Recommendations for early detection.**

- Critically evaluate currently applied early detection methods and their target populations, and select and promote/disseminate those with proven benefit for broader implementation in the EU.
- Promote biological characterization of premalignant disease that progress to invasive and metastatic cancer.
- Stimulate biomarker discovery and development of diagnostic technologies for early detection of lesions that are likely to progress to cancer.
- Support the development of innovative low‐cost devices, methods, and programmes that permit effective early detection with high specificity.
- Develop the concept of prevention screening based on relevant early detection.
- Provide support for their industrial production, testing and validation for use in daily practice.
- Encourage implementation research of early detection programmes, assess participation and analyse factors that affect compliance.
- Analyse clinical effectiveness and health economics of early detection programmes.

### 3.4 Development of new therapies

The number and proportion of academia‐initiated clinical trials (including diagnostics, medical and clinical oncology, radiation therapy, translational associated research, surgery and multimodal treatment) should increase with the specific aim of improving survival and health‐related quality of life, with particular emphasis on precision medicine and age/gender‐specific aspects. New functional and molecular imaging technologies should be evaluated for effectiveness in clinical trials.

Methodologies for predicting treatment outcomes, both positive and negative, are essential for personalized/precision cancer medicine and already receive ample attention in medical oncology, with focus on anticancer agents and immunological treatments [32, 33]. Targeting multiple tumour driving pathways by combinations of targeted drugs applied concurrently or in a specific order may increase the efficacy of treatment by circumventing mechanisms of primary or acquired resistance [34]. Expanding molecular pathology by multi‐omics technologies to identify tumour drivers and conducting high‐throughput functional *in vitro* screens in cells carrying the same lesions might lead to new combination therapies and offer opportunities for drug repurposing [35].

Immunological interventions with checkpoint inhibitors, antibodies, vaccination programmes and cell therapies show ample promise [36, 37, 38, 39, 40]. In addition, developments in radiobiology and radiophysics have boosted innovation in radiation therapies; for example, novel fractionated radiation regimens, use of different sources (photons, protons and light ions), or combination with other treatments offer new perspectives [41, 42, 43, 44, 45]. Surgical treatment is moving towards technologies with improved preservation of organ function and integration with both radiation therapy and medical anticancer treatment [41, 46]. Predicting the best possible intervention will increasingly be guided by big data analyses requiring the contribution of machine‐learning algorithms and computational sciences [47].

Early clinical research delivers proof‐of‐concept outcomes that might have practice‐changing potential. However, it requires further studies to assess their potential value for the health care. For wide implementation in the healthcare system, clear criteria need to be defined for outcomes. Clinical effectiveness has to be assessed in regular practice by collecting real‐life data through implementation research. Survival benefits linked to information on side effects and health‐related quality of life should illustrate the added value compared to current standard treatment. Outcomes of the implementation research should serve as the new gatekeeper when randomized comparative clinical trials cannot be used. Our recommendations for development of new therapies are summarized in Box 4.

Box 4 **Recommendations for development of new therapies.**

- Increase support to academia‐initiated clinical trials (including diagnostics, drug development, radiation therapy, associated translational research, surgery and multimodal treatment).
- Encourage and support research in drug repurposing to find new applications of well‐established and widely available generic medicines.
- Adopt existing and create new innovative investigator‐initiated trial concepts such as Drug Rediscovery Protocol or basket studies, exploring new engagement paradigms with the pharmaceutical industry.
- Support treatment optimization research to identify the optimal dosage and duration of existing treatments, both for the benefit of patients and to guarantee the sustainability of healthcare systems.
- Improve stratification methods of patients using multi‐omics, novel complex multilayer biomarkers based on systems biology models.
- Develop methodologies for predicting treatment outcomes (*in silico* studies).
- Stimulate development and application of new functional and molecular imaging technologies (including radiomics).
- Increase support to already‐established multicentre platforms for early drug development.
- Develop new sophisticated *in vitro* and *in vivo* functional screening methods (e.g. Interspaced clustered regularly short palindromic repeats/Cas9 based in preclinical models, i.e. Patient‐derived xenografts or organoids) to identify new therapeutic paradigms.
- Support the development of academic cell therapy entities (e.g. Chimeric antigen receptor T cells cell production) to boost further innovation in less toxic immunotherapy approaches.
- Promote integration of advanced computational methods (AI, machine learning) with clinical research.
- Structure implementation research in therapeutics to effectively introduce practice‐changing therapies.

### 3.5 Psychosocial oncology, rehabilitation, and survivorship research

Psychosocial oncology, rehabilitation, and survivorship are closely related areas. As the recommendations for each of these areas show substantial overlap, we describe the relevant issues of each first and then provide an overarching set of recommendations (Box 5).

Box 5 **Recommendations for psychosocial oncology, rehabilitation, and survivorship research.**

- Support methodological development for assessment of health‐related quality of life.
- Develop tools to enhance communication with patients and shared decision‐making (e.g. increasing patients' access to their medical records via patient portals, development and testing of decision aids for selecting from available treatments).
- Establish international collaboration for developing survivorship‐specific patient‐reported outcomes in order to monitor the physical and psychosocial health and health‐related quality of life of individuals in the post‐treatment period. This is a prerequisite for establishing effective programmes to address the individual needs of cancer survivors (e.g. return to work, fertility, sexuality, reconstruction surgery, dental health, cognitive functioning, fear of recurrence, etc.).
- Develop, test and implement apps and wearable devices for effective follow‐up monitoring and appropriate interventions.
- Support research to create a comprehensive overview of the negative consequences of a cancer diagnosis and treatment on physical, mental and social health in the short and the long term.
- Develop prediction models for side effects of treatments.
- Support long‐term follow‐up programmes notably for paediatric and young cancer patients to conduct large‐scale, longitudinal, observational studies in distinct cohorts of cancer survivors to better understand their problems and needs.
- Establish and assess outcomes of guidelines to facilitate return to social health, enable reintegration in the workforce and alleviate financial and legal constraints (e.g. life insurance, mortgage).
- Identify health and social inequalities in the cancer survivorship population.
- Initiate research on the economic consequences cancer survivors and their relatives are facing. This should include both direct and indirect costs.
- Evaluate the need for and effectiveness of survivorship care models used in various healthcare systems.
- Conduct research to better understand the causes of differences and discrimination in the survivorship experience between countries and cultures, including financial services such as loans and mortgages.

#### 3.5.1 Psychosocial oncology research

Psychosocial interventions have shown improvement in emotional and social functioning and health‐related quality of life in a large meta‐analysis [48]. Psychosocial oncology is an essential component within the entire clinical trajectory. Technologies to identify patients at risk for psychological distress and to select the most appropriate intervention strategy need further development. Psychosocial oncology can also play a vital role in addressing lifestyle problems as part of prevention programmes, including tertiary prevention as a part of rehabilitation. Psychosocial interventions encompass many research areas such as behavioural science (psychology), epidemiology, public health science, nursing research, sociology and biostatistics.

Communication with patients and relatives is critical, given the new diagnostic and treatment modalities aimed at personalized/precision cancer medicine [49]. Information for the patients will increase in complexity, making it essential to develop tools to ensure that patients fully understand the options available for informed choices in the context of shared decision‐making. Communication with patients is also complicated by the often‐conflicting information patients collect from the internet. With the diversification of treatments and growing number of cancer patients with chronic disease, the demand for information will continue to grow.

Developing guidelines and standards for psychosocial care should be an integral part of implementation research for evaluating programme effectiveness; which patients are offered the interventions and how do they perceive the intervention. A range of demographic, cultural/ethnic, social, clinical and intervention‐related characteristics can influence the relative effectiveness of psychosocial interventions. Thus, it is important to further develop and test tailored psychosocial interventions that fit the needs of specific subgroups of patients as well as individual patients [48, 50]. Research is also needed to identify the psychosocial needs of patients and their families along the entire continuum from diagnosis through treatment and into the survivorship phase.

Although there are a number of well‐researched, psychometrically sound and widely used measures for monitoring the symptom burden, psychosocial needs and quality of life of patients with cancer, additional work is needed [51, 52]. This work could take advantage of available and emerging technologies, such as the use of mobile devices to detect problems at relevant points in time, and eHealth interventions that make psychosocial interventions more accessible to a larger number of patients at lower costs. A promising development is the use of modern test theory, and particularly item‐response theory models and computer‐adaptive testing to refine the assessment of patient‐reported outcomes at the individual patient level.

Despite a large number of publications, further trials are needed to evaluate the health‐related quality of life assessment protocols. Methodological development should focus on new study designs that take advantage of the Internet and wireless acquisition of physical and psychological data. The complexity of assessing health‐related quality of life is increasing with the clinical trials methodology weighting more towards personalized/precision cancer medicine [53, 54]. The latter is a motivation to conduct more research to develop relevant questionnaires.

#### 3.5.2 Rehabilitation research

Rehabilitation is of vital importance for the outcome after cancer treatment [17]. Rehabilitation should focus on three areas: physical, mental health affected by psychological consequences of diagnosis and treatment and, finally, social health (e.g. as influenced by professional reintegration, altered family relationships and financial constraints). High age, comorbidities and frailty [55] are important risk factors for the development of side effects; examples of adverse long‐term effects of disease and intervention are treatment‐induced cardiotoxicity, neurotoxicity, impaired fertility and sexual problems, cognitive impairment and fatigue. Identification and prediction of side effects and psychological complications can assist in the choice of therapy and therefore represent essential research areas. The latter also holds for identification of the needs for supportive care and psycho‐oncological assistance. Research is needed to identify the most effective and efficient intervention strategies for returning to work [10].

For timely detection of complications, long‐term follow‐up is necessary. Patient‐reported outcomes could prove very useful in this regard. Outcomes research should be used for reversed translation to research and design innovative rehabilitation strategies.

#### 3.5.3 Survivorship research

The goal to achieve 10‐year cancer survival for 75% of patients by 2030 poses a major medical, socio‐economic, legal, as well as a political challenge. We need to articulate the most relevant stigmas associated with cancer and convey the message that cancer is no longer a death sentence with cancer survivors having the right to return to a normal life upon recovery.

Cancer survivorship is strongly influenced by the side effects of treatment with a significant impact on patients, the healthcare system and society overall. Long‐term adverse effects have consequences for patients' physical, mental and social health. A review by the former EACS Taskforce on Cancer Survivorship was recently published [56] where survivorship was defined as the phase after active cancer treatment. Survivorship research—the last component of the cancer research continuum and an integrated part of the translational research—has a bearing on the evaluation of multiple outcomes, including symptom burden, functional health, health‐related quality of life and socio‐economics. Information collected from surviving cancer patients may help identify and reduce long‐term side effects of treatment and improve rehabilitation and psychosocial services.

Closer cooperation between clinicians and patients in multidisciplinary and interdisciplinary survivorship research is needed at a Pan‐European level to identify socio‐economic inequalities, including disparities among the EU Member States and in particular the Central and Eastern European (CEE) countries. Reintegration in the workplace and social life, as well as equal rights to take out loans and mortgages, is essential study areas. New legal rules that protect cancer survivors against economic discrimination need to be articulated and proposed to the legal authorities.

The increasing cancer survivorship has initiated discussions about the necessity of specialized cancer survivorship clinical structures within or outside the CCCs, to address the need for infrastructures/facilities for long‐term follow‐up and support of cancer survivors [17]. Long‐term follow‐up is particularly relevant for paediatric and young cancer patients. The development of patient‐reported outcomes surveys tailored to the cancer survivor population is required to ensure that chronic physical and psychosocial health needs can be addressed in an effective and timely manner [57].

### 3.6 Palliative oncology

Supportive care is multidisciplinary and must accommodate the patient's needs. With cancer increasingly becoming a chronic disease following continuous or intermittent treatments, supportive palliative care is crucial until end‐of‐life palliation. Improved therapies translate in life prolongation, but also cause side effects that need recognition, as the overall goal is life prolongation while maintaining a good health‐related quality of life.

Emerging evidence suggests that early integration of palliative and oncological care improves symptom control, health‐related quality of life and even entails a significant life prolongation [58, 59, 60] and higher satisfaction among caregivers [61]. Currently, there is a need to establish supportive care teams or home care teams with expertise not only in caring for the dying patient, but also to address problems, symptoms and side effects associated with palliation among patients surviving for months or years [62]. Our recommendations for palliative oncology are detailed in Box 6.

Box 6 **Recommendations for palliative oncology.**

- Increase research efforts to evaluate the optimal organization of supportive care because emerging cancer treatments often permit a substantial life prolongation.
- Integrate supportive care teams or home care teams into oncological care; implementation should depend on proven clinical effectiveness.
- Promote development and assessment of educational programmes teaching palliative care professionals how to recognize and mitigate potentially life‐threatening side effects resulting from specific treatments (targeted drugs, immunotherapy).

Development and validation of health‐related quality of life assessment methodologies—including psychosocial or existential aspects relevant to patients with severe complications of late‐stage cancer—are urgently needed. Advances in preclinical research might also help to mitigate symptoms, especially in patients with pain or cancer cachexia.

### 3.7 Paediatric oncology

Across Europe, there are more than 35 000 new paediatric cancer cases annually and > 6000 children and adolescents dying from cancer each year. Two‐thirds of the almost half a million childhood cancer survivors in Europe live with the long‐term effects of treatment, which can be severe, affecting their daily lives and socio‐economic participation [63]. While there are interactions across the age spectrum, childhood cancers have a unique set of challenges compared to adult cancers, including the specific types of cancers, the underpinning biology, the clinical pathways, the long‐term physical and psychosocial impact and, crucially, the long‐term support of a sick child by their family.

Childhood cancer accounts for 20% of childhood deaths after infancy and is the leading cause of child mortality from disease in Europe [64]. The European paediatric oncology community already has an extensive track record in the successful delivery of innovative research and clinical strategies from strong collaborative research networks that have markedly improved outcomes. The improvements in the diagnosis and treatment of childhood cancers over the past four decades were built on a strong foundation of cross‐border, multidisciplinary, international research, more recently supported by EU Framework funding programmes.

These established, integrated research and clinical networks are well‐positioned to deliver a further ambitious and integrated programme of international research. The launch of the European Reference Network for Paediatric Oncology (ERN PaedCan) in March 2017 heralded the start of a framework for national healthcare systems to cooperate in the care of children with cancer. International cooperation is essential in the complex and rare disease setting that characterizes childhood cancers. The ERN PaedCan infrastructure enables access to state‐of‐the‐art diagnostics and treatment and facilitates cross‐border exchange of disease‐specific expertise. Further building on this infrastructure will reduce the current inequalities in childhood cancer health care, while also providing a scaffold to integrate research networks (Box 7).

Box 7 **Recommendations for paediatric oncology**

- Support of paediatric cancer projects by investment in research and innovation to specifically combat childhood cancer and reduce disparities.
- Invest in an integrated programme of research to realize the seven key objectives of the SIOPE strategic plan:

- Innovative therapies
- Precision medicine in health care
- Increase biology knowledge of paediatric tumours
- Increase equal access to standard care, expertise and clinical research
- Address the needs of teenagers and young adults
- Improve the quality of survivorship
- Understanding the causes of paediatric cancers and addressing prevention where possible.

In 2015, SIOPE, in partnership with the patient advocate groups Childhood Cancer International‐Europe and Unite2Cure, published a detailed long‐term strategic plan focused on health care and research initiatives to increase survival and the quality of life for children and adolescents with cancer in Europe by 2025 [65]. This strategic plan is evolving to keep pace with emerging innovations and should become part of a European mission to beat cancer. Focus on innovative therapies including precision medicine, next to further research in the biology of paediatric tumours, is an important goal. In addition, equal access to the standard of care and specific attention to teenagers and young adults is an important goal as well as more attention for survivorship issues.

### 3.8 Geriatric oncology

Cancer is a group of diseases mainly affecting individuals at an advanced age, with diagnosis usually above 60 years and death above 70 years. Ageing and cancer are both associated with the accumulation of mutations in DNA [66], and, among other changes, ageing affects the hematopoietic clonal heterogeneity (designated either as ARCH for age‐related clonal haematopoiesis or as CHIP for clonal haematopoiesis of indeterminate significance).

Clones defined by mutations in proto‐oncogenes and tumour suppressor genes accumulate in most tissues with ageing, including skin [67], oesophagus [68, 69], liver [70], colon [71], lung [72] and many others [73]. In the oesophagus, for example, a strong positive selection of clones carrying mutations in distinct cancer genes was identified. With ageing, these clones cover much of the epithelium, with *NOTCH1* mutations affecting up to 80% of cells. Surprisingly, their prevalence is higher in normal tissue than in oesophageal cancers [68].

Widespread positive selection of mutant clones may contribute to tissue ageing by negatively affecting tissue function. Toxic exposures will further increase the mutational burden, as observed in the bronchial epithelium of tobacco smokers [72] and hepatocytes of cirrhotic patients [70]. Furthermore, cells might also become senescent [74]. And although no longer capable to divide, these cells can create an inflammatory environment promoting tumour progression [74].

Currently, many research questions are linked to mutation load and ageing, as well as senescent cells that accumulate during ageing and are associated with a distinct secretory phenotype. These age‐related changes undoubtedly also influence cancer therapy. Therefore, more information is needed regarding the relationship between ageing and cancer (Box 8). We also need to understand how ageing affects treatment feasibility and efficacy and to what extent cancer and cancer treatment accelerate ageing. Targeting senescent cells may become a therapeutic strategy to either prevent or treat cancer as well as to mitigate other chronic diseases (Box 8).

Box 8 **Recommendations for geriatric oncology.**

- Support basic research aiming at understanding the links between ageing and cancer.
- Support clinical research in elderly to optimize treatment.
- Develop instruments, for example frailty scales relevant for oncologic patients, and methods of data collection for assessment of health‐related quality of life in geriatric cancer patients, with an eye for their often‐extensive comorbidities.

High age and comorbidities are regularly exclusion criteria in clinical trials. As a result, we often lack evidence on treatment benefits among older patients. There is a need for clinical trials that analyse dose escalation and de‐escalation, combinations therapies, the impact of comorbidities and the influence on health‐related quality of life (Box 8).

### 3.9 Outcomes research

Outcomes research is essential for assessing the degree to which the goals and objectives of a cancer mission are achieved (Box 9). We need to select robust methods to follow the expected reduction in mortality and increase in long‐term survival. We also need methods to compare outcomes of EU countries and monitor whether inequalities indeed decrease. Outcomes research linked to health economics is fundamental for priority setting with an important role for patients/patient organizations. Lead‐time bias due to early detection and overdiagnosis of nonlethal cancer has to be taken into consideration when survival benefits are analysed. Interpretation of trends in cancer patient survival is indeed challenging and never straightforward [75]. There is a need to define time frames for short‐term (5 years) and long‐term goals. Increases in the 10‐year survival rate among patients diagnosed through 2030 will be impossible to assess until 2040.

Box 9 **Recommendations for outcomes research.**

Different domains of cancer need definition of distinct outcome parameters. No outcome will be relevant to all. The main domains are cancer therapeutics and prevention.

1) Cancer therapeutics

*a) Short‐term*

- Assess clinical effectiveness of innovations—in combination with health economics analyses as a ‘gate keeper’ before implementation into the healthcare system.
- Monitor the percentage of patients in clinical trials and compare outcomes for patients in and outside clinical trials.
- Study short‐term overall survival to mitigate effects of lead‐time bias and possible overdiagnosis.

*b) Long‐term*

- Study 5‐ and 10‐year overall patient survival to mitigate effects of lead‐time bias and possible overdiagnosis.
- Study 5‐ and 10‐year cancer overall mortality and cancer‐specific mortality (rate of death of cancers in the population, stratified by age and gender, and other relevant risk factors).
- Assess all‐cause mortality (although new treatments may not reduce all‐cause mortality, all‐cause mortality should be used as an endpoint to ensure that harms of the new treatment do not affect other causes of death).
- Determine health‐related quality of life after 5, 10 years and longer.

2) Prevention

*a) Short‐term*

- Assess population receptivity to prevention interventions.
- Assess the potential impact of intervention programmes on the prevalence of behavioural risk factors for cancer, such as smoking, alcohol consumption, obesity (of the whole population) as a function of intervention programmes.
- Monitor the percentages of patients and individuals included in behavioural research and in prevention trials or other studies aiming at reducing the cancer burden.

*b) Long‐term*

- Assess trends in cancer incidence, cancer mortality and overall mortality.
- Study effects of cancer prevention strategies on mortality in the population.

Outcomes research has been a missing element in large parts of translational studies (see the chapter on infrastructures). CCCs should contribute with quality‐assured and consistently structured clinical registries to monitor assessment of clinical effectiveness of implementation, including documentation of reproducibility of research outcomes in clinical practice. Outcomes research is also needed to demonstrate the effectiveness of prevention initiatives. The OECI has started programmes for the development of outcomes research, and health services research, within its constituent CCCs. In addition, the German Cancer Research Consortium has an expanding clinical database bringing together the information of eight of the leading German CCCs.

Implementation of personalized/precision cancer medicine requires scientific evidence on an increasing number of subgroups based on new molecular pathology/genomics diagnostic technologies. Even for common tumours, the large number of subgroups will offer challenges similar to those in studies of rare cancers; for future studies to be informative, international collaboration will often be a prerequisite, so that patient numbers are sufficiently large for reaching statistically robust conclusions.

Outcomes research should be classified into short‐term and long‐term assessment for cancer care, including therapeutics (Box 9). Benefits of prevention, on the other hand, can be meaningful to assess only as a long‐term effect, although short‐term outcomes may guide quality assurance and acceptability in the population. Valid outcomes research requires high‐quality data (see above) and the ultimate outcomes are cancer incidence, mortality and overall survival of cancer patients.

Many population screening programmes to detect and prevent cancer early may result in healthy individuals undergoing unnecessary tests and treatments [76]. Early detection screening, such as prostate and breast cancer screening, increases the recorded incidence of cancer [77, 78]. Prevention screening such as cervical and colorectal cancer screening increases the incidence of precursors (cervical intraepithelial neoplasia for cervix and colorectal polyps for colorectal cancer) but decreases the incidence of invasive cancer. Efforts should be made to improve the prognostic value of cancer screenings and reduce the burden for the individual: to do more good than harm—‘less tests less treatments’.

### 3.10 Health economics

New possibilities for cancer prevention, diagnosis and therapies usually come from findings resulting from public and private investments in medical research [16]. Their numbers rise rapidly, making informed choices necessary. This leads to an increased interest in clinical and cost‐effectiveness research. The impact on population health depends on what one pays for in the different European healthcare systems. Current data reveal significant differences in inputs and outputs, and this is reflected in the performance measures [79]. Health economics studies the unavoidable choices between different alternatives when resources are limited. European healthcare systems differ with respect to available resources for cancer care and how those resources are used, but they share the same objectives of improving outcomes for cancer patients. Development and sharing information for making the best use of available options given limited resources for cancer care are a common interest.

The objective of a mission‐oriented approach to cancer research in Europe is to improve health outcomes for cancer patients through the development and introduction of new methods for prevention, early diagnosis and treatment of the disease, using surgery, radiotherapy and cancer medicines. Health economics includes the study of the efficiency and equity of resource allocation to and within cancer care. A key point in the translational research process is when decisions are to be made about pricing and reimbursement for the introduction of a new method or drug in clinical practice in different countries. Decision‐makers, including public payers, clinicians and patients, should have accurate information about clinical effectiveness, costs and overall value of the new method/drug to decide about use and payment. These decisions are not only important for improving outcomes for patients and healthcare efficiency, but also for the research community in prioritizing investments in the development of new methods.

Often robust data on clinical effectiveness and value for patients of new methods compared to existing alternatives are lacking. For example, the number of new cancer medicines increases fast. But there is rather limited information from clinical trials on outcome parameters, as compared to alternative treatments [80]. Follow‐up studies in clinical practice have serious shortcoming in terms of data on patient characteristics and methodology and thus not fulfilling their potential to contribute to evidence generation and improvements over time [81]. The latter is a problem that cannot be mitigated by more sophisticated health technology assessment methods. The potential consequence is the introduction and regular use of methods and medicines that have little or no value, or a delay in the introduction of new treatment regimens that do improve outcomes for patients.

At present, we have also incomplete information about cost‐effectiveness of resources used to treat cancer [82]. Data are lacking about the resources spent for different types of cancer care and for different groups of patients and how this affects outcome.

Therefore, decision‐makers, including public payers, clinicians and patients, need better information about the potential clinical effectiveness and value of the new method in order to make decisions about their use and reimbursement. Health economics needs to be included as an integral component of the translational research pathway. Therefore, research including aspects of SES (socio‐economic status) is important in order to promote equal access to cancer care. Without public reimbursement through taxes or public health insurance, appropriate cancer care is not affordable for the general public. The pricing and budget impact of cancer medicines on the healthcare system poses a particular challenge and requires close monitoring of objective benefits and costs and patients should be involved in health economics research at all levels. Our recommendations for health economics implementation are summarized in Box 10.

Box 10 **Recommendations for health economics.**

- Make the collection of data for an assessment of cost‐effectiveness a mandatory part of all clinical research projects aimed at developing new preventive or therapeutic methods within the cancer mission.
- Evaluate already existing methods (in fact deferred maintenance) as a validated reference.
- All applications for clinical research grants should include a statement of how the project will contribute to the objectives of the mission, and a plan for how the impact should be assessed.
- Support the development of a database carrying the relevant information to appraise cost‐effectiveness of preventive and therapeutic innovations.
- Support the advancement of methods that assess the social value of cancer care beyond aggregate gains in length and quality of life of patients, that are relevant for decisions about allocation of resources for cancer; severity of disease condition; necessity of intervention; prevalence of the condition; and impact on caregivers and dependents of patients.
- Install a task force that continuously evaluates and reports on the cost‐effectiveness of new innovations in prevention and therapeutics, as information to healthcare systems to decide on adoption and reimbursement. The task force should also assess if the cancer mission research programme achieves its objectives.

### 3.11 Big data and computational science

EU‐wide population databases will be indispensable for answering some of the questions listed above, including comparative research between geographically distinct regions in Europe. This requires consistency in institutional clinical registries that need to be based on standardized patient records with genomic/molecular marker information, providing opportunities for specific studies such as Outcomes Research and Health Economics Research as outlined above. Assessment is needed of the value, validity and reliability of voluntary patient‐reported data uploaded to a single EU digital centre and its compatibility with privacy and ‘droit d'oublier’ requirements. There is also much work to be done on how to aggregate detailed datasets for research purposes, while guaranteeing patient anonymity. Work is also needed to develop AI paradigms for mining data to identify new correlations and meaningful algorithms (improvements in predicting response, relapse and side effects). Sophisticated diagnostic methods and algorithms to interpret them are needed to select the most promising cancer therapy for individual patients (e.g. to avoid the commonly observed selection of resistant clones [83, 84, 85]. Our recommendations on big data and computational science are summarized in Box 11.

Box 11 **Recommendations for big data and computational science.**

- Stimulate introduction of AI/machine‐learning approaches in multiple areas: image analysis, whole‐genome sequencing, patient‐reported outcome information, clinical record datasets, lifestyle parameters, prevention measures and early detection.
- Define the core data records that should be collected from every patient, complemented with predefined disease‐specific and patient‐specific records, on the assumption that certain data stay in the treating institution unless that patient gives permission for wider use.
- Explore whether patient‐initiated data sharing provides an option to create large well‐accessible and reliable datasets without violating existing privacy rules.
- Offer practical training courses focussed on acquiring new computational skills relevant for research and clinical care.
- European data protection policies need to prevent the misuse of data without restricting the use of data.

## 4 Patient empowerment

The primary focus of patient empowerment is on improving the healthcare systems, so that the patient is at the centre of shared decision‐making.

The cancer mission aims at covering the entire research continuum. By definition, translational cancer research has a focus on patients and individuals at risk and strives to improve all aspects associated with the consequences of a cancer diagnosis. In traditional research, patient participation was largely limited to being the subject of research. Currently, there is a significant cultural shift that increasingly ensures that real‐life experiences of patients are considered when determining priorities in research areas [86].

Patients that actively participate in research focused on unmet needs develop increased self‐confidence, and a more robust advocacy voice, making them feel more empowered, valued and respected. Early patient involvement in research offers opportunities for identifying and influencing research questions and defining meaningful study endpoints. Patient empowerment, as far as cancer research is concerned, is mostly related to unmet needs of patients.

Comprehensive cancer centres integrate care, prevention, research and education enabling innovation in multidisciplinary care. Patient perspectives are important, and since assigning priorities to projects is unavoidable, patients should be represented in CCC boards, while CCC leadership also establishes formal interactions with patient organizations.

The integration of patient advocacy in the full spectrum of childhood cancer research and multidisciplinary care is exemplified by the partnership between ERN PaedCan and the CCI‐Europe, the primary patient and survivorship organization in Europe. CCI‐E representatives are core members of the Network's Oversight Committee, as well being intrinsic to the implementation of the ERN's objectives at the national level.

It will be necessary to involve patients' representatives in the governing bodies of all consortia and infrastructures mentioned earlier in this article. Similarly, patients and patient organizations should have a role in the different project areas suggested above (Box 12).

Box 12 **Recommendations for patient empowerment.**

- Support primary and secondary prevention with a focus on individuals with modifiable risk.
- Involve patients and patient organizations in prioritizing therapeutic research areas.
- Support rehabilitation research, and research focussing on health‐related quality of life issues (supportive care, psychosocial oncology, palliative care and survivorship) including patients and families for shared decision‐making.
- Involve patients and patient advocacy organizations in prioritizing research areas in outcomes research and health economics. This should also include assessment of the socio‐economic impact on patients and their families (/households/relatives/dependents and caregivers), and the identification of patient groups particularly vulnerable to impairments of their socio‐economic situation due to cancer and cancer care.
- In areas where research focuses on how to decrease present inequalities, patients and patient organizations should be enabled to play a pro‐active role.
- Shared decision‐making should ensure that all medical and social consequences of a cancer diagnosis are considered.
- Education is a prerequisite to reach the goals of the mission. Both European Cancer Patient Coalition (ECPC), Association of European Cancer Leagues and SIOPE have extended educational programmes for patients, relatives and the public. Increase collaborations with CCCs and consortia of research centres will be necessary to further expand the educational activities.
- Communication and diffusion of information are vital to bring science and technology to society and to emphasize their importance for generating science‐driven and social changes that impact the lives of patients. The mission governing body, cancer patient organizations, national cancer societies, universities and hospitals, policymakers as well as the press, should broadly disseminate the information.

## 5 Specialist education

Education must cover all components of the cancer research/care/prevention continuum and be accessible to researchers and cancer specialists from all EU countries to reach the goals of the mission on cancer [87]. Leading European cancer organizations [EACR, EACS, European CanCer Organisation, ECPC, European Molecular Biology Organization (EMBO), EORTC, European Society for Medical Oncology (ESMO), European Society of Surgical Oncology, European Society Radiotherapy and Oncology, Federation of European Biochemical Societies and SIOPE] regularly organize conferences and support educational courses. In addition, Cancer Core Europe organizes an annual Summer School for Translational Cancer Research, the OECI focus on the comprehensiveness of cancer care and the ECPC on education centred on patients and their relatives. Trainings in cancer prevention are currently organized irregularly and would benefit from a more systematic approach, both reaching out to medical and public health professionals.

An inventory of educational activities within the EurocanPlatform project revealed an impressive amount of educational activities in 23 participating cancer research centres (https://cordis.europa.eu/project/id/260791/reporting). Making courses accessible to students and professionals from all Member States will increase knowledge and promote networking. Exchange of researchers will foster new research collaborations in consortia of cancer centres. Furthermore, the twinning of centres can greatly help in disseminating expertise and establishing a critical research culture. Implementing our recommendations as outlined above and summarized in Box 13 will decrease inequalities across EU countries and facilitates capacity building. Specific educational programmes targeting the next generation of leaders will support sustainability and increase interaction between research centres as exemplified by Cancer Core Europe.

Box 13 **Recommendations for specialist education.**

- Establish recurrent educational and scientific conferences prepared by the organizations mentioned above.
- Organize theoretical training courses.
- Create a new European comprehensive culture of education, training and lifelong learning.
- Extend the reach of educational courses by arranging participation also through the internet.

## 6 Inequalities in research

Emphasizing the link between cancer outcomes and research activities, the EC recognized that increasing the quality and quantity of research capacities is needed to improve outcomes for cancer patients with specific attention to high‐risk individuals in the Member States (https://ec.europa.eu/programmes/horizon2020/sites/horizon2020/files/SPH_VisionPaper_02062016.pdf) [88].

To reduce disparities with the primary aim to improve patient survival as well as the health consciousness in the Central and Eastern EU region, Prof Miklós Kásler, Minister of Human Capacities, Hungary, took the lead in bringing 21 countries together. His initiative resulted in the foundation of the CEEAO, within which institutions join forces in fighting cancer in a population encompassing 260 million people. In January 2020, the governing council and the scientific advisory board of CEEAO were elected at its general assembly in the Hungarian Parliament. The organization aims at harmonizing cancer control plans in the region with a focus on cancer care, prevention and education. As an example of a well‐functioning consortium within the region under the umbrella of CEEAO, the wide‐ranging, coherent activities of Central‐Eastern European countries within the Central‐Eastern European Breast Cancer Surgical Consortium are worth mentioning. The ERN PaedCan has already achieved at least one ‘node’ per country in Central/Eastern Europe for development of paediatric oncology.

Despite the presence of excellent basic and clinical research in some areas, translational research activities largely suffer from insufficient funding and limited collaborative activities in the Central and Eastern EU region [89]. For example, a dedicated cancer research fund is not available in many countries. In addition, the number of clinical trials (in particular early clinical trials and investigator‐initiated trials) is lagging in this region [90]. Hence, innovation in prevention, early detection and treatment could have a significant impact on cancer incidence and survival in many Central and Eastern European countries. To this end, tighter collaboration between clinical and basic research activities should be enabled primarily by strengthening the scientific activities of accredited cancer centres.

Our recommendations for addressing inequalities in cancer research are summarized in Box 14. As noted above, the OECI's and EACS's accreditation and designation programmes should serve as primary quality control of translational cancer research and its integration into high‐quality patient care in Europe. CCCs accredited for their care, research and education should play a central role in fulfilling the aims of the cancer mission. In parallel, the accreditation programme intrinsic to the ERN PaedCan is driving quality for research for cancer in children and young individuals. These entities constitute the powerhouses not only of high‐quality cancer research in Europe; they also provide the best opportunity and model for a strong interaction between research and multidisciplinary health care, a pivotal element to ensure that innovations benefit patients.

Box 14 **Recommendations for addressing inequalities in cancer research.**

- Strengthen Central‐Eastern European cancer centres with effective utilization of OECI's Accreditation and Designation programme, and EACS's Designation of Excellence (DoE) programme via collaboration with the CEEAO.
- Extend and strengthen the Paediatric Cancer Expert Reference Network to be accessible to children with cancer throughout Europe. Promote concentration of paediatric cancer research and care where feasible.
- Support cancer research activities that address region‐specific issues in cancer care, prevention, research and training within Europe.
- Open dedicated calls for proposals in the Central‐Eastern EU region to decrease inequalities in basic, clinical and translational cancer research.

**Fig. 4:**
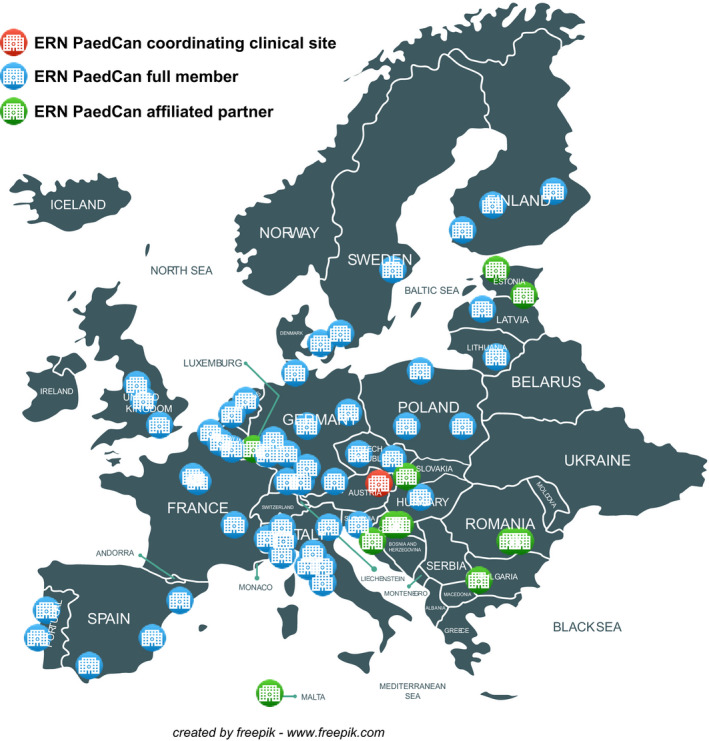
ERN PaedCan Network for paediatric cancer. Distribution of members over Europe.

Out of the 40 OECI accredited centres, 22 hold the CCC designation and 18 are designated as clinical ‘Cancer Centres’ (CC), which represent recognized, high‐quality clinical centres, although with significantly less research output. Inequalities become immediately evident by the geographic distribution of these accredited cancer centres because out of the 40 OECI accredited centres, there is only one OECI accredited CCC and five accredited CCs in the Central‐Eastern EU region (Fig. 2). The ERN PaedCan unites 57 Full Members from 18 countries and a further 12 Affiliated Partners from eight countries (Fig. 4).

## 7 **Relationship between the cancer mission and Europe**'**s Beating Cancer Plan**

The decision to support European cancer activities with both a European Beating Cancer Plan (https://ec.europa.eu/health/non_communicable_diseases/cancer_en ) and a cancer mission is timely and strategically relevant. There are apparent inequalities both within and among EU countries concerning cancer treatment, or care, and cancer prevention. For example, access to early detection programmes, advanced diagnostic methods, immunotherapy, precision medicine, state‐of‐the‐art surgery, radiation therapy, functional/molecular imaging or rehabilitation is highly variable. The EU project European Network for Cancer Research in Children and Adolescents (https://siope.eu/activities/eu‐projects/encca/) also demonstrated significant inequalities in paediatric oncology between EU countries. The Europe's Beating Cancer Plan will be valuable to coordinate national cancer plans to make better use of evidence‐based cancer treatment/care and prevention. The latter will mitigate inequalities by supporting national programmes for equal access to cancer patients and survivors.

## 8 Concluding remarks

A comprehensive translational cancer research approach that is focused on personalized/precision medicine and covers the entire cancer research‐prevention‐care continuum has the potential to achieve in 2030 the goal of a 10‐year cancer‐specific survival for 75% of the patients diagnosed in EU Member states with a well‐developed healthcare system. Expected effects of primary prevention on incidence and mortality is a more long‐term goal to be assessed by age‐standardized mortality monitoring. Concerted actions across this continuum that spans from basic and preclinical research through clinical and prevention research to outcomes research, as well as the establishment of high‐quality networked infrastructures will pave the way not only to clinical innovation, but also to the mitigation of economic and social inequalities across European countries.

Here, we propose the establishment of three types of infrastructures focusing on translational research, clinical and prevention trials, and outcomes research. These infrastructures, embodied in CCCs or CCC‐like entities, will provide researchers with access to a critical mass of patients, biological materials and technological resources, bridging research and health care. The latter will warrant that future scientific and social innovations benefit cancer patients across the healthcare systems in Europe.

We prioritized 13 research areas to achieve a balanced research portfolio, namely: basic and preclinical research; primary prevention; early detection for prevention and treatment; development of new therapies; psychosocial oncology, rehabilitation, and survivorship research; palliative oncology; paediatric oncology; geriatric oncology; outcomes research; health economics; big data and computational science. We have worked together to provide recommendations for each of the above areas; these recommendations will be, in our view, important for achieving key targets. We also offer suggestions as to how to strengthen patients' empowerment, improve specialist education, and decrease present inequalities in cancer research within the EU.

Meeting key objectives will require further harmonization of EU and national priorities and policies, improved research coordination at the national, regional and EU level, as well as more efficient and flexible funding mechanisms. It is also crucial to ensure the sustainability of trans‐border infrastructures and networks, for example through long‐term support directly by the EU, or other schemes to which Member State countries commit. It will require political will and perseverance to bridge the gaps in science, society and policy that affect cancer treatment and care [91]. Science policy is often developed in isolation [91]; therefore, it will be crucial to engage policymakers and to ensure that all the relevant stakeholders along the entire research–care–prevention continuum speak with a single voice to provide evidence‐based advice to inform policy [25, 91]. In addition, careful forward planning will be pivotal to ensure a successful outcome.

A concerted cancer science policy in Europe is an unmet need [91]. Appointing a policy board with multiple competencies will be necessary to identify the best strategies to implement the comprehensive range of activities necessary to accomplish the mission goals.

## Conflict of interest

In the past 5 years, Dr. Baumann attended an advisory board meeting of MERCK KGaA (Darmstadt), for which the University of Dresden received a travel grant. He further received funding for his research projects and for educational grants to the University of Dresden by Teutopharma GmbH (2011–2015), IBA (2016), Bayer AG (2016–2018), Merck KGaA (2014–2030), Medipan GmbH (2014–2018). For the German Cancer Research Centre (DKFZ, Heidelberg), Dr. Baumann is on the supervisory boards of HI‐STEM gGmbH (Heidelberg) and is also member of the supervisory body of the Charité University Hospital, Berlin. Dr. Baumann, as former chair of OncoRay (Dresden) and present CEO and Scientific Chair of the German Cancer Research Centre (DKFZ, Heidelberg), was or is responsible for collaborations with a multitude of companies and institutions, worldwide. In this capacity, he has signed/signs contracts for his institute(s) and for the staff for research funding and/or collaborations with industry and academia, worldwide, including but not limited to pharmaceutical corporations like Bayer, Boehringer Ingelheim, Bosch, Roche and other corporations like Siemens, IBA, Varian, Elekta, Bruker and others. In this role, he was/is further responsible for commercial technology transfer activities of his institute(s), including the DKFZ‐PSMA617 related patent portfolio [WO2015055318 (A1), ANTIGEN (PSMA)] and similar IP portfolios. Dr. Baumann confirms that none of the above funding sources was involved in the preparation of this paper.

Carlos Caldas is a member of AstraZeneca's iMED External Science Panel, a member of Illumina's Scientific Advisory Board and a recipient of research grants (administered by the University of Cambridge) from Genentech, Roche, AstraZeneca and Servier.

Outside the scope of this work, Caroline Dive declares research funding/grants received from: AstraZeneca, Astex Pharmaceuticals, Bioven, Amgen, Carrick Therapeutics, Merck AG, Taiho Oncology, GSK, Bayer, Boehringer Ingelheim, Roche, BMS, Novartis, Celgene, Epigene Therapeutics Inc, Angle PLC, Menarini, Clearbridge Biomedics; personal honoraria for consultancy and/or advisory board has been received from: Biocartis, Merck, AstraZeneca and Illumina.

Carolina Espina and Joachim Schüz state: where authors are identified as personnel of the International Agency for Research on Cancer/ World Health Organization, the authors alone are responsible for the views expressed in this article, and they do not necessarily represent the decisions, policy or views of the International Agency for Research on Cancer/ World Health Organization.

Peter Nagy is supported by the 2019 Hungarian Thematic Excellence Program (TUDFO/51757/2019‐ITM).

Josep Tabernero reports personal financial interest in form of scientific consultancy role for Array Biopharma, AstraZeneca, Bayer, BeiGene, Boehringer Ingelheim, Chugai, Genentech, Inc., Genmab A/S, Halozyme, Imugene Limited, Inflection Biosciences Limited, Ipsen, Kura Oncology, Lilly, MSD, Menarini, Merck Serono, Merrimack, Merus, Molecular Partners, Novartis, Peptomyc, Pfizer, Pharmacyclics, ProteoDesign SL, Rafael Pharmaceuticals, F. Hoffmann‐La Roche Ltd, Sanofi, SeaGen, Seattle Genetics, Servier, Symphogen, Taiho, VCN Biosciences, Biocartis, Foundation Medicine, HalioDX SAS and Roche Diagnostics.

All other authors declared no conflict of interest.

## Author contributions

All authors contributed to the preparation of the manuscript.
