# Corrigendum to: Towards a cancer mission in Horizon Europe: recommendations

**DOI:** 10.1002/1878-0261.12801

**Published:** 2020-10-02

The authors of “Towards a cancer mission in Horizon Europe: recommendations” (Mol. Oncol. 2020 Aug; 14(8): 1589‐1615. PMID: 32749074) [1], have decided to update Fig. 4 and the related legend, to clarify that it visualizes information about the distribution and numbers of ERN PaedCan network, using as vector image a product of artwork, and not a political map. This change does not affect any discussion, or conclusions made in the article.

**Fig. 4:**
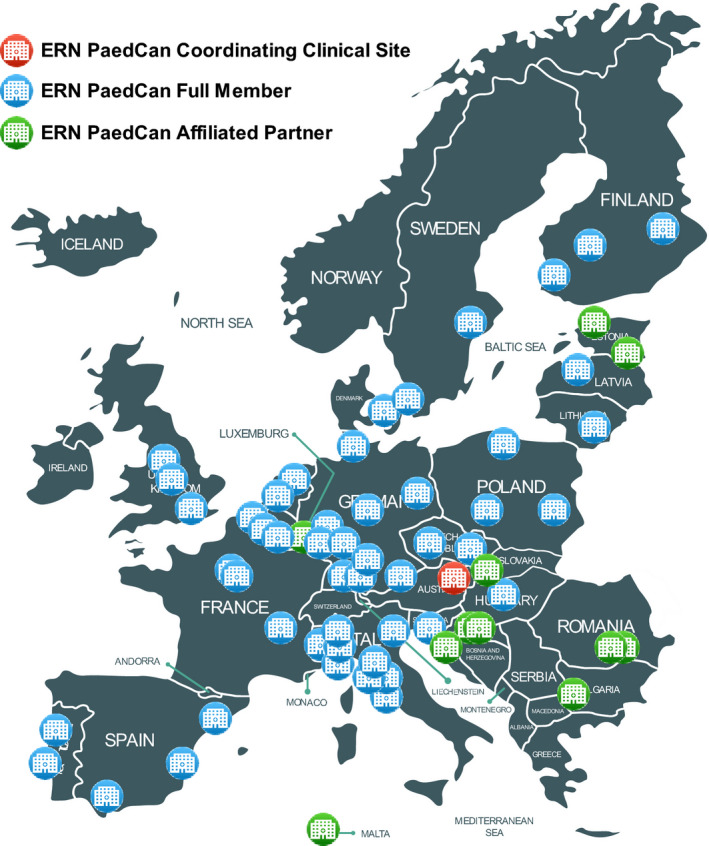
Distribution of members of the ERN PaedCan network for pediatric cancer. Information is visualized as an illustration and not as a political map.
